# Developing Microbial Solutions to Advance Sustainable Agricultural Productivity

**DOI:** 10.1089/ind.2018.29144.pzo

**Published:** 2018-08-01

**Authors:** Paul Zorner, Larry Walker, Donald Marvin, Janne Kerovuo

**Paul Zorner:** I'll start with a few comments to introduce this panel. I've spent a good portion of the last 40 years trying to integrate microbiology and agricultural production. Unfortunately, as I think most of us realize, until very recently the performance and adoption of these potentially powerful tools has been limited, at best. But it is amazing to me, the sophisticated new tools that are emerging in many biotechnology sectors which are allowing the community of innovation involved in agriculture to place these new tools in this space and change the performance characteristics that have thus far limited the adoption of and confidence in microbial products in agriculture.

EDITOR'S NOTE: This article was derived from a Breakout session at the 2018 Biotechnology Innovation Organization's World Congress on Industrial Biotechnology, July 18, in Philadelphia, Pennsylvania.

We can not only provide growers with consistently performing and cost-competitive products that I think can compete with conventional chemistry. But truthfully, I think we can replace conventional chemistry. Biology in its most basic form is much more powerful than conventional chemistry in terms of complexity and its potential for what can be functionally accomplished.

Last year, Larry Walker, an old friend, came to me and proposed we document these innovations and advanced technology. And the result was a special issue of *Industrial Biotechnology.* It is a great special issue put together by a remarkable team of people. This is not going to be the only issue we do like this. I would like to recruit and encourage everybody to come to us to submit papers. It is a very easy, fast process. We want to create a conversation, not just here today but a broader conversation over time.

Using the term “agricultural probiotics” is a way to make the science more relatable to the general public. I don't know about my colleagues here, but when I talk about what we're doing I find it best to use terminology that people see on TV every day about prebiotics and probiotics and their impact on human health, and thus can more readily make the connection to what is happening in agriculture. We want to convey that science is showing that a healthy microbiome is just as important to a crop as it is to you and I. You're sitting here, with 10 times as many bacterial cells as human cells, and a genome that is 8 times the size of your genome, and you're absolutely dependent on that extra metagenomic functionality. Crops are no different. It is clear that the yield potential of crops is a function of both its own genes and a properly structured metagenome associated with the crop.

So our session today is intended to relay what we have learned in putting this special issue together, where we at *Industrial Biotechnology* are headed with respect to further issues, and to document the continuous stream of innovation and the remarkable people who are driving it.

Speaking of remarkable people, we have three here today: Larry Walker, Janne Kerovuo, and Don Marvin. And we're going to start with Larry, who is of course the 2018 winner of the BIO [Biotechnology Innovation Organization] Legacy Award. So congratulations Larry. I spent a lot of hours with Larry in a lot of different places in the world and you are never going to find a more passionate and dedicated scientist. Larry spent many years as a professor of biological and environmental engineering at Cornell University. He calls himself a retired professor, but I haven't seen the retired part yet! Larry is also co-editor-in-chief of *Industrial Biotechnology*.

**Larry Walker:** It is a real pleasure to be here with you today. I am here in my capacity as co-editor-in-chief of *Industrial Biotechnology.* To explain why we did this issue and how it relates to my interest and background. I am an engineer. I am not a molecular biologist. I am a not a soils and crop specialist. Some people think that's good and some people think that's bad.

We would all agree that the breakthroughs in the life sciences are creating unprecedented opportunities to engage sustainability challenges. I feel that a lot of engineering principles are important to what we do in industrial biotechnology space, but you don't have technology unless you are meeting a need. Technology innovation is about meeting a need: providing food, providing water, providing ipads, you're meeting a need.

To this audience, that is apparent. But to some of my academic colleagues, you have to keep reminding them that it's not just about the science. It's how the science is deployed to meet challenges. The other thing we tend to underestimate is the experience base. This panel here today, we have a fantastic experience base to tap into.

My experience and my interests have been shaped by my research activity during my 36 years at Cornell. Part of my goal was to introduce engineering into the life sciences. I did a lot of work with anaerobic digestion, composting, biocontrol, molecular ecology, metabolism, nanobiotechnology, systems biology and systems engineering. I did this all with an aim: to understand how do we manipulate and engineer complex microbial systems. If you think about it, in the past we have focused on pure culture: a single microorganism that is going to do it all. But in nature, microorganisms collaborate. They work together to carry functions.

Of course, part of this for me is driven by science fiction. The Martian talks about bacterium producing potatoes, or terraforming. But terraforming on Mars or in outer space is far in the future. But we need to look at how to do terraforming on Planet Earth.

The future, for me, is providing microbial solutions for agriculture. And that solution also involves engineering consortium. It has been picked up in a number of places, including the magazine Economist, which argues that we need people who understand and are better able to manipulate the crop biome. Part of my interaction with Cornell involves people looking to control environment agriculture. For the Northeast, that is a big deal because people are interested in hydroponics to produce fresh produce. What is it about microbial flora in hydroponic solutions that confer pathogen resistance? What is going on in that system.

Paul and I spent some time in India together, where we've worked with local farmers using an old-fashioned method for cultivating peppers and tomatoes. This farm in India had all of these vats mixed with soil with run off from the sugar crop with dairy manure, and it was producing a cocktail that they were spraying on their peppers. The peppers looked beautiful! How do we understand these types of systems, document who the players are, and how do we replicate and improve on them?

As we start looking at these systems, we know why people look at pure culture. Pure culture is predictable and controllable. But when you start talking about consortia, you're talking about cocktails of microorganisms, you depart from linearity in terms of behavior of the system, heterogeneity of the material the microorganisms is growing in, you have to deal with uncertainty. It is a dynamic and complex process. As an engineer, I get excited about working in that space.

We have unbelievable tools out there that we can begin to tap to help us understand and model crop microbiomes and look at to what degree we can manipulate them. It will really require collaboration to exploit these tools and begin to move this into the realm of predictability.

I'll wrap up by discussing the Journal. There is a need for us to move on a third or fourth green revolution. My colleagues here would agree on that. What are the needs? Of course we talk about higher yields to feed the planet. But in this world, we are also concerned about more efficient nutrient utilization. P and K are finite resources, and how do we begin to make better use of those resources? This includes more efficient utilization of water, better control over plant pathogens and other pests. How do we move away from pure culture to do that? And improving the quality of the food that we produce? The taste?

All this has to be done with the public in mind. For those of us working in the biotechnology, we know we need to be careful about how we label things, how we talk about them.

The major component that we are looking at is a system where the behavior of the system is more than the sum of the parts. How do we begin to think about manufacturing microbial products that are dispersed? How do we begin to package and handle these products for shipping? Some of you may remember in the lignocellulose area, the big business model change was not to ship the enzymes from the enzyme plant, but to produce them at the biorefinery. It is a licensed technology. This wasn't a scientific breakthrough, this was a business breakthrough.

How do you keep cell viability? How do you validate that what you proposed to introduce in the microbiome, that those microbes that are there and working? You need a systems perspective on microbial solutions. But there are a number of challenges that we need to work out in a holistic way.

And where do we go from here? The journal will continue to seek and publish content in this area. We believe this is a very important area for the future of agricultural biotechnology. Paul has agreed to join our editorial board as an Associate Editor, and we're going to be reaching out to you folks to give us original research, industry reports, and commentaries. We want to be the voice in this area.

Let me wrap up by saying that a lot of what has driven me is the notion that we have sustainability challenges that we need to address. I think microbial solutions have great potential. We need to preserve the environment. We need to collaborate and produce good teams to produce good science to solve these challenges.

**Zorner:** Next up is Janne Kerovuo, vice president of research and discovery from NewLeaf Symbiotics. I've known Janne for a long time as well. We both worked at Diversa together 10 or so years ago.

**Janne Kerovuo:** NewLeaf is a five-year-old company focused on beneficial microbes for agriculture. We are located in St. Louis, Missouri, close to the Danforth Plant Science Center, which is a perfect place for a company like ours. We have close proximity to field trials, a lot of greenhouses, and growth chambers from Danforth Center that we can use as we need.

The strategy NewLeaf has taken is to focus on methylotrophic plant-associated microbes for the benefit of plants. We are focusing on identifying and developing the best fit M-trophs for any crop we are working on—we are focusing on corn and soy at the moment—as a biocontrol agent or biostimulant, as a seed treatment, in furrow, or even in foliar applications. We think of these microbes as bio-complements; they will work with and boost existing chemistries and existing germplasm and traits.

So why focus on M-trophs? They are very robust and can colonize all plants. They can colonize the phyllosphere, rhizosphere, endosphere, and there is also evidence of certain M-trophs living inside the plant cell, which is quite unusual if you think about it. They are very rich with known plant-enhancing traits, like the production of phyto-hormones and 2,3 butanediol, etc. There are a lot of known traits for enhancing plants.

In addition to that, they help seeds to germinate better. If you're coating your seeds with M-trophs, you get a more uniform germination. We have also shown that when we mix certain M-trophs with certain agricultural inputs that are going on the seed as a seed treatment, we can achieve colonization of the entire plant.

We have also seen that certain M-trophs enhance the photosynthetic traits of the plant. We see enhancement in chlorophyll content and enhancement in photosynthetic efficiency.

M-trophs are unique in that they utilize methanol secreted by the dividing plant cells as an energy source. There is no real energy cost for the plant in hosting M-Trophs.

For gram-negative microbes, these are fairly easy to “productize.” They are desiccation-tolerant, they are compatible with many agricultural chemistries, and we can grow them to high titers.

Our research and discovery cycle: we source from the wild; we are focusing on wild grasses and legumes. We take the samples to the laboratory and perform our proprietary microbial isolations. We genotype and fully sequence all the microbes that we isolate. This is where we differ from others in my mind—the lead-generation rates with M-trophs are very high. We are not running in vitro plant growth-promotion, growth chamber plant growth-promotion or greenhouse plant growth-promotion assays as much as others might be. Instead, we perform compatibility screens and plant colonization screens. When I think about what is required from the microbe as a product in the end, it has to be tolerant of desiccation and compatible with ag chemistries. So we are thinking about the product and what are the phenotypes of the microbe required for the end product. And then all the data goes to our computational bioinformatics platform called Prescriptive Biologics Knowledge Base.

In one example, we randomly took 65 M-trophs from our collection based on genomic diversity. We wanted to cover as much diversity out of our collection as possible and I could only afford to do 65 strains out of 1,200 strains at the time—so that's testing about 6% of our collection directly in the field. We coated these strains on elite soy with seed-applied insecticides, seed applied fungicides, and a control for a multi-location trial.

We saw 6 bushels per acre increase with our best-performing strains. Looking at the phylogeny of the collection at the time, and looking at the top 12 and the bottom 12, there was no phylogenetic localization of winners. They were pretty much all over the phylogeny. However, with our computational bioinformatics platform, when we ran an association analysis with the winners and the losers, we started seeing genes that are enriched only in the winners and never in the losers and vice versa. So that gives you a very powerful comparative genomics and gives you marker genes that you can go back in the collection and look at what other strains we might have with these marker genes.

We are not only working on bioyield and biostimulants. We performed multi-location, multiyear studies in corn root worm mitigation. Results showed that we are on par with trait and chemical insecticide. We are not necessarily saying that we are going to replace the trait or chemistries. What this gives farmers is a very good opportunity for integrated pest management. We think of these tools as a biocomplement. If you apply our M-troph with the trait, the lifespan of the trait should be significantly increased because you are bringing a second mode of action and resistance is less likely to build.

We launched two products this year, which is pretty good for a five-year-old company. Terrasym 401 is a soy biostimulant and Terrasym 402 is a biostimulant for peanuts. The soy product is a seed treatment and the peanut product is an in-furrow application.

So right now we are at the 25,000 liter scale, and as we speak we are scaling up to 130,000 liter. Current commercial products are freeze-dried and we are not implementing spray drying technology. And like I said about product viability, we have demonstrated one year of stability and one-plus years of shelf life.

**Zorner:** Next up is Don Marvin, the only one of my fellow panelists that I haven't worked with, but we have talked over the phone a bit lately. And one thing that came up is that rising tides lift all boats. It's a big market and I think there is a lot of potential for cross collaboration between companies like ours. Don is going to speak about Concentric but also speak a bit about what it takes to create a company like this and make it viable and sustainable to get across that valley of death and have an impact.

**Donald Marvin:** I'll just talk a little bit about Concentric and our view of this emerging and very exciting biological space within the overall ag industry. At Concentric, we produce biological and plant nutrient crop inputs comprised of mixed communities of microbes and essential nutrients. The company has three locations. We started out in Montreal. Montreal is our Technical Center of Excellence, comprising 20,000 square feet, where we do a lot of our technology development, product development and manufacturing of microbial-based products. Our US operations are located outside of Denver, Colorado at a 35,000 square foot facility. This is our head commercialization office where our executive leadership team sits, and it is our second manufacturing facility. With our recent acquisition of ATP Nutrition in Winnipeg, Manitoba, we have a 25,000-square-foot EPA-registered facility to manufacture plant nutrients and do R&D in that space.

Our fermentation platform is patented and very sophisticated. It enables us to combine multiple strains of bacteria and fungi into synergistic solutions. It took us about five years to develop this fermentation platform. The microbial combinations are compatible with each other, but you would not normally find them coexisting in nature. All of the strains in our products that are in the marketplace today come from the food industry. So the general regard is that they are safe, they are already in foods that we consume every day. It is truly an artificial consortium that was put together in a way that, through the cofermentation process, very interesting things happened. We designed the strains as a kind of biological factory that contributes to soil health and helps plants uptake nutrients more efficiently.

In the end, our goal is like everyone else: Help our partners increase yields and give farmers more bang for their buck by making nutrients work more efficiently. In many cases, farmers' input costs actually drop because our biologicals help optimize plant nutrients, again enabling growers to spend less for inputs.

We have two products in the market today. SYNERGRO^®^, which is the live cell formulation containing ten microbes—seven bacteria, and three fungi. It is registered in 22 states across the United States as a soil amendment and all across Canada as an amendment as well. It was one of the first consortia ever registered. It is used by many customers in the specialty ag space, particularly high-value crops.

Our second product in the market was just launched this year: SYNERGRO FREE^®^. It is produced through a very similar fermentation process, but at the end of the fermentation we remove all the microbes and concentrate the actives. Why did we do that? Because we didn't want to ship water all over the place. And most importantly, we wanted to be at the right price point for broad acre crops. This particular product was in three years of field trials with many different proprietary micronutrient packages to be applied in-furrow, as a seed coating, as well as foliar applications.

With our recent acquisition of ATP Nutrition in Winnipeg, we are now starting to work on a new class of biological crop inputs that will combine our biologicals with micronutrients already produced by ATP to really drive the full genetic potential of the crop.

All of this really contributes to our vision for agriculture and the Second Green Revolution. We look at the entire growing environment, the plant microbiome, if you will—the seeds, the plants, the root systems, the soil, the surroundings—and that's what we focus our business on. What does healthy soil really mean? I think people struggle to define it. But there are some folks out there at the Soil Health Institute who came out with a detailed measurement system to determine what soil health is all about. There are 19 measurements indicating soil health. I think the subject is going to continue to evolve in the ensuing years. You're not going to solve this thing overnight given the richness and variety of microbes in soil and their associated micronutrients.

We are focused on soil health because healthy soils and rich microbial activity are better able to deliver nutrients to plants. At the same time they retain water better. They more efficiently store carbon to reduce the effects of climate change. We are scientists first and foremost. Our products are underpinned by rigorous R&D and it is our mantra. But, we know that to really ensure that our products will perform, you have to conduct many field trials as some of my colleagues have also discussed. This is really a biological product, and so I always get suspicious when people talk about win rates of 85% or yields of 20–30%. I'm always really skeptical of that, only because this is a biological system and it is an imperfect system.

We also touch the economic factors that concern growers. We have worked within agriculture's existing business models because we think that is the easiest and fastest way to deliver these new tools to the grower. I think a lot of people in our industry and other companies are promoting new, disruptive business models as sort of a du jour way to describe their businesses these days. Frankly, I say good luck to them because they are going to put financial resources and human resources into changing a model in the industry that works quite well today and has worked that way for 50 to 60 years. Rather, we focus on the new types of inputs that are going to deliver incremental value to the grower and to make them money. We plan to offer a broad range of new products including the roll out of new products next year.

**Zorner:** Before I open it up for questions, I will start with a comment and a couple of questions of my own. I don't know how many people noticed, in the Senate Farm Bill that was just passed, there is base language in the bill, in which they are promoting and incentivizing quote/unquote healthy soil for growers and starting a national program to demonstrate best practices in terms of creating healthy soil. This is not just healthy soil defined by the conventional things: aeration, nutrients, moisture, etc, but also recognizing that microbial soil health is very important and developing ways to measure that is critical. I belong to a group called Environmental Entrepreneurs, and we went and helped advocate some of this base language. It was amazing to me that sitting U.S. Senators actually understood microbial soil health. I was shocked and impressed. And I think it's an indication that people are really realizing that this is extremely critical. FDR once said: A nation that destroys its soils destroys itself. We're on the right side of history here as an industry in terms of what we are trying to do and a lot of these remarkable technologies are really going to help with that.

Janne, you mentioned biocomplements. You have to work with existing biology and Don, you inferred the same. But is it working with existing biology or is it reconstructing holes made by the overuse of conventional practices: chemicals, tillage, a variety of things? Are there things missing that the crops need, that if you can replace them, you can stimulate the plant to express this natural genetic potential?

**Kerovuo:** With our corn rootworm data, we believe that what happens there is that the M-troph we are applying really triggers something in the plant. I'm not just talking induced systemic resistance, we believe that there is something else going on that is totally untapped territory in our mind. So, the answer is yes.

**Zorner:** Plus they are complex genomes. Don, I think you alluded to the same thing. The first time I talked to you, you talked about SYNERGRO FREE product. The microbes, in your case are initial product, are like little factories producing the actives. Can you speak to what you think is going on there? Are the metabolites themselves acting directly or simply stimulating the plant to do something?

**Marvin:** That's the $64,000 question. Obviously, it's an interesting one. We look at soil in the first Green Revolution back in the 1940s with Normal Borlaug and NPK, and we've been using that for many, many years now. It's really wrecked a lot of soil out there. Now you need to build that soil back up. And so, you can do that microbially and through essential nutrients if there are deficiencies of essential elements and nutrients. We offer those products through ATP. But also I think it's the microbial composition of that soil as well. Your question of what is going on is a good one. We went down a cell-free route if you will for those reasons. For business reasons, not necessarily scientific reasons. But, SYNERGRO FREE actually contains the special metabolites that are produced during that co-fermentation process. And those metabolites, as you might expect, have multiple modes of action. And those multiple modes of action stimulates the plant to increase yield across many different cropping systems. Some of those modes of action we understand pretty well. From unlocking phosphorous and other nutrients in the soil other modes of action that are produced vis-a-vis those many different, interesting metabolites that are produced, that we are still looking to understand. And we are careful about how we talk about those because this is a regulated product. It is important to not cross that barrier. We spend a lot of money on R&D, on really understanding that mode of action and really understanding how we can optimize the fermentation process, which is really the jewel of our company. We can swap strains in and out of that fermentation process that throw off different interesting products from biostimulation to biofertility to biocontrol. We have a pretty robust new product pipeline in all three of those areas vis-a-vis that fermentation platform. It's an intriguing area. It's one that I think is going to be pretty exciting as we go forward over the next four to five years.

**Walker:** We don't have a good feel for metabolic fluxes in these systems. So think about what we do: We work with two or three comfortable microorganisms, we document their behavior and response. What we really need to understand is how microbes are interacting. When microbes are interacting, they turn on different pathways. They are not always having the same pathways that you have in pure culture. So one of the things that we are missing is understanding those fluxes within complex soil, complex structures. And so, I would say we are probably missing a lot.

We are also missing the microorganisms that we have not cultured. And I think a lot of the molecular ecology studies over the last 15–20 years have shown that we're missing major players in the soil and how those players are interacting with other microorganisms.

**Zorner:** When I was at Diversa, we looked at that a lot. And what we found is that many organisms are culturable if you actually grow them as complex systems. What we found, is that when we went out and made our collections, and then grew these organisms up as a community, we actually got them to culture. It's genomic cross-talk. There's a lot to be learned there.

***Audience question:*** *There is a lot of negative reaction to phrases like genetically engineered, genetically modified. These are scary terms for the public. So I was wondering, is this industry working on a coherent language for this emerging technology that does not evoke such an emotional response. Even the term biodiversity, which one would think would have a positive reaction has had negative views.*

**Zorner:** A lack of understanding breeds a lack of confidence. Phrases like herbicide-tolerance are not warm, fuzzy. As a young scientist, I was on a team that was looking at salt-tolerance and drought-tolerance. Those are more warm and fuzzy.

Larry, I'm going to toss that question to you to start, because I know you have some thoughts.

**Walker:** I would just add that this group, the industrial biotechnology community, its members are very sensitive to labeling. We saw what happened, how things got out of control around the GMO debate. And part of that was terminology, and not getting ahead in terms of public education. What you're seeing with some of the players, is that they are being careful. One of the things that we want to make sure we do in the journal, is have a discussion about how do we engage the public in the discussion. Social needs. You can come up with the best product in the world and it goes nowhere because people don't accept it. So we have to have the public involved in an informed dialogue. And we have to do that on multiple circuits.

**Zorner:** Don, you talked about business models. Agriculture is a local, trust-based environment. Trust is very important. How do we engender trust?

**Marvin:** It's a good question. When I speak to the investment community, the laymen, the consumers, the farmers, I usually try to break it down into a lexicon that people understand. I will say our products are probiotics for plants, at the end of the day. People get that. It's in the lexicon of today.

I also think that, in our case, our microbes are not genetically derived. They come in food and we consume them every day. I think there is an opportunity here to work in a collaborative fashion with all my colleagues here but also the other companies in this space to really determine what those communication factors are. How do you communicate this to the public? At the end of the day, they are eating the products that we treat with our products. I think there is a huge opportunity to work in collaboration, not as competitors but as collaborators. It's wide open to really work together to make that happen.

**Zorner:** There is a trend now of people not just worrying about what they eat, but what their food eats. How that food is grown. We have to engage in this public dialogue.

***Audience question:*** *Looking at Canada versus the US, is Canada a similar markets for your products in terms of composition, acceptance, and uptake?*

**Marvin:** Obviously the larger market of the two is the United States, which is why we set up our operations outside of Denver. The markets themselves are quite similar in many regards. When we got started, we introduced SYNERGRO into the specialty ag space, and we did that because we wanted to learn from the market. We wanted to learn from the product how we could improve it. And quite frankly we had not come up with the right formulation to scale it for broadacre crops at the right price point.

The similarity in Canada is that a lot of those high-quality produce crops are grown at 100 acres or less. And so, to me, the similarities are interesting. One is outside here in the United States. The other is under glass all across Ontario and other provinces of Canada. And they ship those products all over the place. In terms of broadacre crops, I'm really amazed that you can grow soybeans in North Manitoba as well as corn. The genetics have been so fine-tuned for those particular crops it boggles my mind. But in terms of broadacre crops in Canada, a lot of our customers are canola growers and wheat. And now we're moving those products here in the United States, focusing again on the oilseed crops. Not necessarily going to the Iowa farm belt where there are 80 million acres of corn and that many acres of beans as well, because I think everyone looks at that market and they say, “Gee, if I could just get a tenth of a percent of that market, it would really be a terrific company.” But I think they fail to understand that those channels are controlled by the big guys. And no matter how great your technology or how sexy it is, you can't get in those channels very easily. So I think we focus on the upper plains on wheat, canola, sunflower and other oil crops where they are underserved by the big guys. We have great products that drive the full genetic potential of those crops. And we've proved that across Canada.

We look at both markets differently, but synergistically.

**Zorner:** I have another question for Janne. It relates to the two different approaches, and I'm purposefully making a division between what Don and Concentric does and what Janne does. Don talked about ten organisms—seven bacteria and three yeast. And here you are Janne, concentrating an R&D strategy on not just one organism, but one particular genus. Would you comment on your strategy and why you are not looking at a broader range?

**Kerovuo:** We're focusing on this one particular genus of bacteria because it works. It has really high lead-generation rates. But we are also not just working on one strain, we are mixing M-trophs with other M-trophs. We are also mixing our M-trophs with rhizobium inoculants. So we're not just working necessarily with one. What we've seen is that, even when we apply just one M-troph on a soy seed and look at the microbiome after the M-troph has colonized the soy plant and increased the yields and the microbiome of those plots to control plots, we have changed the microbiome of the plant. Something is happening there. If I look at the composition of the M-trophs in the control plot, the total number of M-trophs is actually higher on the control than in the M-troph treated plots that are performing: meaning we have put the right M-troph as a seed treatment and are getting a yield bump.

**Marvin:** We got on the metabolite route pretty early. I spent a lot of my career at pharmaceutical companies, and there it is all about finding the active or the drug-like molecule and then packaging it to deliver it to the right receptor in the body. So we went down that route early at Concentric. If you stop and think about it, our microbes really come from the food industry yet we're introducing them in a live-cell formulation into the rhizosphere of the plant itself. So you have to ask yourself: Do they survive? Do they colonize? And it's a very good question. I would say in some cases they probably do and in some cases they probably don't. But what is carried along with those microbes is the supernatant, which really contains the metabolites themselves. I think it's a very interesting conversation to have: The rhizosphere and whether you colonize it. The rhizosphere is a pretty nasty place with all those bugs, particularly if you are now introducing an artificial consortium that originated in the food industry and that really isn't as robust as what you might see in the rhizosphere.

We focus on the metabolites themselves and how they are taken up into the plant. How they might stimulate the existing rhizosphere and look at them as prebiotics rather than probiotics. I don't think we have all the answers, let alone all the questions. And it's going to take time.

***Audience question:**** How have you seen metabolomics evolving over the last 20 years and how do you apply that to what you're doing?*

**Kerovuo:** If you want to do metabolomics right, as Larry would say: You have to stick with the real system. If you start teasing that apart, I don't think there's any reason to do metabolomic analysis because it's not going to be what you would see in situ.

So how do you do metabolomic analysis in a soy field in Iowa? That's a tough thing to do. If you take that sample to the lab and do the metabolomics there, it's different than what it would be in the field. There lies a huge challenge for not just metabolomics but all transcriptomics studies and all 'omics studies. You would somehow have to do it in situ and that would be tough.

**Walker:** We tend to go from fundamentals in the lab and build studies. And part of that is beginning to develop model studies with attributes in the crop microbiome, including the physical structure you would work with and begin to do those types of consortium experiences there. Where you would have some control over the interactions and begin to do the measurements. We had done that with some of the anaerobic and aerobic digestion processes that we worked with to find that middle ground. But not too far away from the fundamentals that it has no meaning in the application.

I can go back to some work I did with aerobic degradation. That's where I know mixed cultures the best: Compost. Composting is a very dynamic process. Early on we found that if we focused our attention on just the spatial dimension as opposed to try to look at the radial dimension, we could simplify our models. We also found that if we set up our sampling protocols properly with the techniques available for molecular ecology, we could pull samples and get a picture of the dynamic response of the microorganism. So we could pick up the responses to pH, to temperature, to water content. Those are the same factors you would use with soil. But if was controlling the dimension and being very specific about the parameters.

We started there, and then our molecular biologist got us started on these molecular ecology tools for say, “Who's there?” and quantitative work. Engineers are hung up on numbers. So how do we go in there and get numbers on these systems and really start to do population dynamics and build metabolic models.

I'm not saying it's going to be easy—and I tend to think this is something universities and experiment stations should do because it's going to take awhile to get results.

***Audience question:**** Can you talk about the challenges around stabilizing cell viability in relation to the application?*

**Kerovuo:** It does matter how you ferment or produce your microbe for the efficacy for certain strains. So you have to be really careful there that you hone in early on production and the formulation, so you don't just have a viable strain in the end, you have a strain that really provides best benefits for the plants. It's been shown that even though the microbe is the active ingredient that will start growing with the plant and provide benefits, there's evidence that the way it is produced does matter. Two different production methods might create very different results in the field with the same microbe.

**Walker:** In some of the work that we have done with decomposition, we can see how small changes in the initial inoculum sends you off on a different trajectory in terms of the behavior of the system. That should not be surprising for us, because these systems are highly non-linear. One of the attributes of non-linear systems is that small perturbations can take you off on a different trajectory. Uncertainties may be caused by the non-linearity. I could have three reactors running side-by-side, first two would track identically, while the third would go off in a completely different trajectory. Why? We set them up the same way.

**Zorner:** It is complex. I spent a good portion of my career on fermentation. There are many ways to do this. At Locus, we produce our microbes locally, keep them in a cold chain and deliver within hours to a couple of weeks of production. It's worked well for us. There are folks who have demonstrated that they can produce viable, strong products with conventional distribution and fermentation. Concentric is certainly one of them.

**Marvin:** Our SYNERGRO product is a live-cell formulation. It has an 18-month expiration date on it. It is stable at ambient temperatures so it doesn't have to go on a cold chain. There is not activation, so when you introduce that product to the field or the plant, they are ready to go now. You don't have to wake them up. We recommend that you don't put it in direct sunlight or freeze the product. But it is pretty stable at ambient temperature.

You would think that that SYNERGRO FREE would have a long shelf-life and indeed it does. We're still compiling a lot of the stability data, but even after you take out all the microbes and you have the actives left, you still have a very rich broth. And if you're not careful you'll contaminate it. So we actually put a preservative in that particular product to maintain its consistency while it's being shipped and used by the grower.

**Zorner:** The science is great, but innovative companies are dependent on their people. I'd like each of you to comment on where you get talent? What skills do you look for? Do you look in ag, or people outside of ag who can bring new inventions inside of agriculture?

**Marvin:** I grew up on a farm, so I think understanding farmers' economics at his or her level is important as you put together a new product. I've learned a few things. Most importantly: if it doesn't work in the hands of the farmer, it doesn't work. So you might as well pack up and go home. Customers are very central to us. You have to understand what the customer is doing. What are his or her farming practices and how do you insert your solution into those farming practices? And, at the same time, you have to understand the lexicon.

We look to bring in people with a strong agricultural background, particularly on the commercial front. On the R&D side, not as much. Like Janne's, our technology is pretty sophisticated, like what you would find in any life science or biotechnology company. And so I think understanding technology is important on the research side. But as you move in the field trials you absolutely need some ag expertise.

Where do we find these people? All across the country. You really need to look everywhere. Major universities. If we can pluck them out of other ag companies and they want an entrepreneurial experience, we'll go after them.

I try to hire people who are smarter than me, give them the resources and then let them do their jobs. I get out of their way.

**Walker:** You've heard the word collaboration quite a bit here today. And that collaboration should go between industry and universities. One of the things I was very fortunate to do because of BIO, is have this connection to great people. A whole group of people who I could bring to Cornell and interact with my students and faculty. My students are interested in these activities, but they don't know what the opportunities are. They don't know what these companies are working on. So I would ask you all to come and engage with universities, because we need to find that next generation of young people to carry the torch.

**Kerovuo:** As I mentioned, we are close to the Danforth Center, so we get talent from there. We get some talent from the former Monsanto. However, in general, we get people around the country. Where we struggle a little is to find software developers for agriculture. They tend to go to tech companies and get paid a lot more than we can afford to pay them. That said, several members of my bioinformatics team chose agriculture because they wanted to make a difference. They didn't want to be coding a useless app. Those are the people you want to hire. These are young people. Like 23–25 year olds. I'm really happy to have them.

**Walker:** If I can just add to that: You want not just people with computational mathematical skills, but people who have a systems perspective. When I talk with companies who are really out there pushing an agenda, they say, “We can find the breeders that we need. What we can't get is people who understand how to bring breeding along with other technologies and science into an integrated system.” Having people who understand systems dynamics and systems modeling is an important part of it.

